# Hepatic SIRT6 Deficiency Accelerates Female-Specific Aging Through SULT1E1-Mediated Estrogen Depletion

**DOI:** 10.3390/ijms27157039

**Published:** 2026-08-05

**Authors:** Yonghui Liu, Ziliang Zhang, Tingting Wang, Xiuyan Yang, Qiufen Zhang, Xinyi Liu, Xuefeng Lu, Jian Zhang, Li Feng

**Affiliations:** 1 School of Pharmacy, Ningxia Medical University, Yinchuan 750004, China; qliuyonghui@163.com; 2 Department of Pharmaceutical and Artificial-Intelligence Sciences, Institute of Medical Artificial Intelligence, Shanghai Jiao Tong University School of Medicine, Shanghai 200025, China; 3 State Key Laboratory of Drug Research, Shanghai Institute of Materia Medica, Chinese Academy of Sciences, Shanghai 200031, China

**Keywords:** SIRT6, sexual dimorphism, SULT1E1, estrogen metabolism, liver aging, cellular senescence, MDL-800

## Abstract

Sexual dimorphism is a fundamental feature of aging, yet the liver-centric mechanisms underlying sex-specific aging trajectories remain incompletely understood. SIRT6, a NAD^+^-dependent deacetylase, is a master regulator of genome stability, metabolic homeostasis, and longevity. However, its cell-type- and sex-specific functions in aging have not been fully characterized. Here, we generated hepatocyte-specific *Sirt6* knockout (HKO) mice of both sexes and evaluated longitudinal lifespan, comprehensive metabolic profiling, hepatic histopathology, and transcriptomic profiles. Female HKO mice exhibited an acceleration of aging and a 17.60% reduction in median lifespan, characterized by severe systemic gerometabolic decline, visceral adiposity, and advanced metabolic-associated fatty liver disease (MAFLD). In stark contrast, aged male HKO mice displayed an enhanced catabolic state and lipid-clearing phenotype via the compensatory reprogramming. Mechanistically, hepatic *Sirt6* deficiency in females exclusively hyperactivated the expression of estrogen sulfotransferase SULT1E1, resulting in reduced circulating estradiol levels. This hormonal collapse triggered a self-amplifying pathological triad of de novo lipogenesis, genomic instability, and cellular senescence. Crucially, pharmacological SIRT6 activation using the small-molecule activator MDL-800 suppressed *Sult1e1* expression, restored estrogen homeostasis, and successfully rescued the gerometabolic phenotypes in aged female mice. Collectively, these findings demonstrate that hepatic SIRT6 regulates female lifespan and healthspan by safeguarding estrogen homeostasis, defining the druggable SIRT6-SULT1E1 axis as a sex-stratified therapeutic target against age-related metabolic decline.

## 1. Introduction

Aging is characterized by progressive physiological decline in organismal integrity, accompanied by increased susceptibility to metabolic disorders, cancer and other chronic diseases [1,2,3]. Crucially, aging trajectories exhibit profound sexual dimorphism: females generally display longevity advantages but distinct vulnerability to metabolic and autoimmune disorders compared with males, who show earlier-onset cardiovascular and neoplastic risks [4,5]. Endocrine regulation contributes substantially to these differences. Estrogen confers female-specific metabolic and anti-inflammatory protection, contrasting with androgen-associated risks in males [6,7]. Aging further drives sexually divergent alterations in body composition, insulin resistance, and hormone dynamics (e.g., insulin-like growth factor-1 (IGF-1) and sex steroids) [8,9]. Notably, accumulating evidence indicates that tissue-specific metabolic and epigenetic mechanisms also participate in shaping sex-dependent aging trajectories [3,10,11,12]. However, the molecular basis underlying these sexually divergent aging phenotypes remains incompletely understood.

Caloric restriction (CR) robustly extends lifespan and healthspan across species [13] through nutrient-sensing pathways including sirtuins, AMPK and mammalian target of rapamycin (mTOR). Notably, its anti-aging benefits are sexually stratified, with estrogen signaling emerging as a key mediator of these protective effects in females [14,15,16]. Estrogen signaling mediates CR’s protection via multiple mechanisms: optimizing gonadal receptor ratios for follicular survival [17], driving female-preferential metabolic reprogramming to enhance insulin sensitivity [18], and potentiating anti-inflammation and mitochondrial-stabilizing actions [19,20]. The liver and adipose tissue serve as pivotal sites for orchestrating these sex-specific benefits of CR. Sirtuins, a family of nicotinamide adenine dinucleotide (NAD^+^)-dependent deacetylases, function as core integrators of these nutrient-sensing responses [21]. Within this family, SIRT6 has emerged as a central longevity regulator through its roles in DNA repair, genomic stability, metabolic control and inflammation resolution [22,23].

Accumulating evidence indicates that CR upregulates hepatic SIRT6 levels [24], and systemic SIRT6 overexpression extends lifespan by restoring energy homeostasis [25], Critically, global SIRT6 deficiency causes severe premature aging and early postnatal lethality in both sexes in mice and monkeys, indicating that SIRT6 is indispensable for organismal survival irrespective of sex [26,27]. However, emerging evidence suggests potential intersections between SIRT6 and sex-specific physiology. For instance, early gain-of-function of SIRT6 studies show lifespan extension primarily in males [28], and recent work has linked SIRT6 overexpression to male liver-specific chromatin aging countermeasures [29]. SIRT6 can regulate estrogen function by stabilizing estrogen receptor α (ERα) to enhance estrogen sensitivity [30] and preserving ovarian collagen homeostasis [31]. Despite these clues, previous studies investigating SIRT6’s critical metabolic and homeostatic roles, such as in glucose metabolism [32], obesity [33,34,35,36] and fatty liver [37,38], predominantly utilized mixed-sex cohorts or lacked direct sexual comparisons. Consequently, whether SIRT6 functions in a sex-dependent manner within specific metabolic tissues remains largely obscured, and its potential cell-type-specific actions in driving divergent sex-specific aging trajectories have not been characterized.

To bridge the gap and define the exact role of hepatocyte SIRT6 in sex-specific aging, we generated hepatocyte-specific *Sirt6* knockout (HKO-*Sirt6*) mice of both sexes and subjected them to comprehensive long-term phenotypic and transcriptomic analyses. Our findings establish hepatic SIRT6 as a master regulator of sexually dimorphic aging trajectories. We show that hepatocyte *Sirt6* deficiency selectively drives a female-biased pathological aging phenotype, manifested as a significantly shortened lifespan, increased serum IGF-1 levels, exacerbated DNA damage (γH_2_AX accumulation), increased susceptibility to metabolic-associated fatty liver disease (MAFLD) and neoplasia, and adverse body composition shifts. Mechanistically, comprehensive transcriptomic profiling revealed that hepatocyte SIRT6 loss causes an aberrant induction of the estrogen sulfotransferase SULT1E1 exclusively in females, creating a localized “estrogen sink” that depletes active estradiol and triggers lipogenic activation and cellular senescence, whereas males adapt via compensatory thyroid hormone signaling pathways. Furthermore, pharmacological activation of SIRT6 using the small-molecule activator MDL-800 [39] successfully reversed these female-specific gerometabolic phenotypes primarily by suppressing *Sult1e1*. Collectively, this work identifies hepatic SIRT6 as a critical guardian of estrogen-metabolic homeostasis that selectively protects females from accelerated aging, establishing a novel, cell-type-specific mechanism underlying sexual dimorphism in liver aging.

## 2. Results

### 2.1. Sex-Specific Impacts of Hepatocyte-Specific Sirt6 Deficiency on Lifespan and Aging Phenotypes

**Figure 1:**
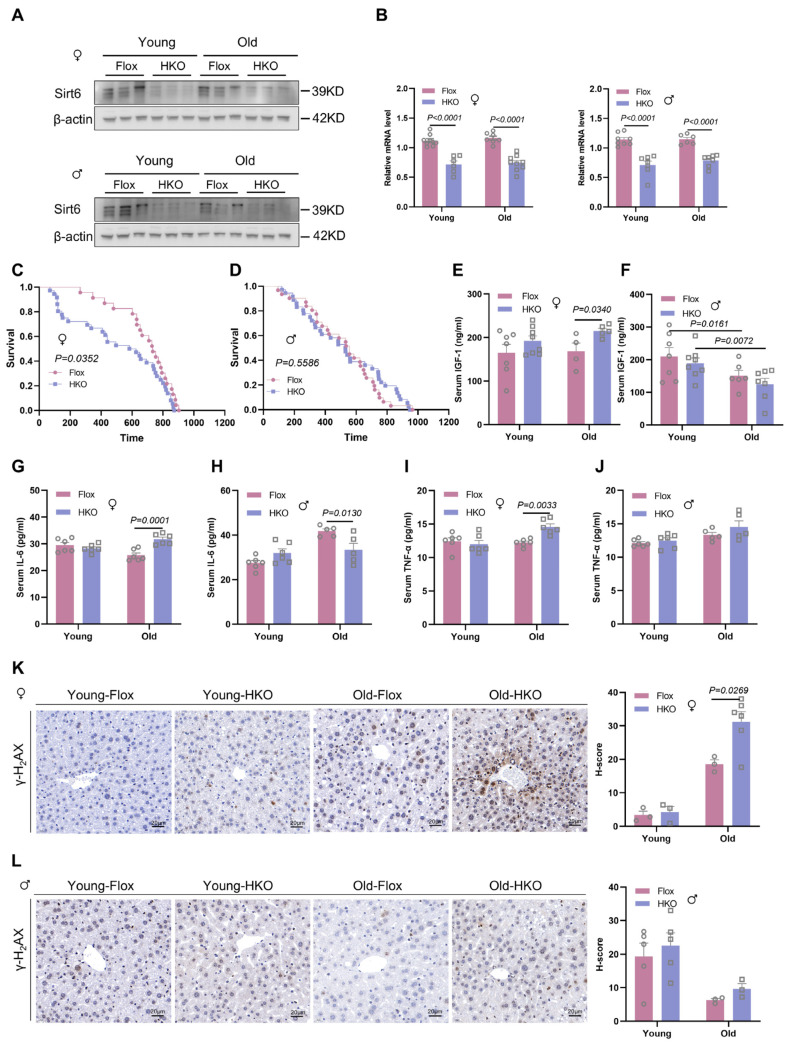
Hepatocyte-specific *Sirt6* deficiency drives female-biased lifespan reduction and accelerated ageing. (**A**) Hepatic Sirt6 protein expression in young and aged *Sirt6*-floxed control (Flox) and hepatocyte-specific *Sirt6* knockout (HKO) mice of both sexes. Representative Western blots with β-actin as the loading control (*n* = 3 biologically independent mice per group). (**B**) qPCR analysis of hepatic *Sirt6* mRNA levels normalized to *Gapdh* (*n* = 6–9 mice per group). (**C**,**D**) Kaplan–Meier survival curves showing a significant reduction in lifespan in female (**C**) (Flox: *n* = 23, HKO: *n* = 36; log-rank *p* = 0.0528, Gehan–Breslow–Wilcoxon test *p* = 0.0352) but not males (**D**) (Flox: *n* = 31, HKO: *n* = 36; log-rank *p* = 0.5586, Gehan–Breslow–Wilcoxon test *p* = 0.9500). (**E**–**J**) Age-associated serum biomarker profiles in mice: IGF-1 (**E**,**F**), IL-6 (**G**,**H**), and TNF-α (**I**,**J**). (**K**,**L**) Representative immunohistochemical images (left) and quantitative H-scores (right) of γ-H_2_AX foci (DNA damage marker) in female (**K**) and male (**L**) liver sections. Scale bar, 20 μm. Quantification based on ≥3 independent mice per genotype (5 fields/liver). ♀: Female, ♂: Male. Data are shown as mean ± SEM. *p* values were determined by two-way ANOVA with Sidak’s multiple comparisons test (**B**,**E**–**L**).

To investigate the sex-dependent roles of hepatic SIRT6 in aging, we generated hepatocyte-specific *Sirt6* knockout (HKO-*Sirt6*) mice by crossing *Sirt6*^flox/flox^ mice with albumin-Cre mice (Supplementary Figure S1A). Genotyping confirmed equivalent hepatic Sirt6 ablation in both sexes at the protein (Figure 1A) and mRNA levels (Figure 1B), with no detectable deletion in white adipose tissue (WAT) or skeletal muscle (Supplementary Figure S1B).

Survival analysis revealed a sex-dependent effect of hepatic *Sirt6* deficiency on lifespan. HKO-*Sirt6* females exhibited a 17.60% reduction in median lifespan compared with Flox-*Sirt6* controls (median lifespan: 734 days vs. 605 days, respectively; log-rank *p* = 0.0528; Figure 1C). The Gehan-Breslow-Wilcoxon test, which is more sensitive to differences occurring earlier during the survival period, revealed a significant difference between genotypes in females (*p* = 0.0352). These findings suggest that *Sirt6* deficiency accelerates premature aging specifically in females. In contrast, males showed no mortality difference (*p* = 0.5586; Figure 1D). Consistently, pooled analysis of both sexes masked this effect (*p* = 0.6978; Supplementary Figure S2A), further supporting a female-specific acceleration of aging. Comparison of the maximum lifespan (the age of the last surviving mouse in each group) of Flox- and HKO-*Sirt6* mice showed no difference for females (Flox: 903 days; HKO: 873 days) and males (Flox: 895 days; HKO: 878 days).

Mechanistically, hepatic *Sirt6* deficiency triggered age-dependent sexually divergent IGF-1 responses (young: 3–7 months and aged: 12–30 months). Two-way ANOVA identified a significant sex × genotype interaction in aged mice (*F* _(__1, 18)_ = 4.80, *p* = 0.042; Supplementary Table S3). Aged-HKO females displayed elevated serum IGF-1 levels (Figure 1E), a key regulator of longevity that is inversely associated with lifespan [40,41]. In contrast, males showed an age-dependent decline in IGF-1 that was unaffected by genotype (Figure 1F). Pro-inflammatory responses similarly exhibited sex-specific dysregulation. For IL-6, significant sex × genotype interactions were observed in both young (*F*_(1, 20)_ = 5.242, *p* = 0.033) and aged mice (*F *_(1, 20)_ = 22.0, *p* = 0.0002) (Supplementary Table S3). Consequently, aged HKO females showed elevated IL-6 levels (+23.4%, *p* = 0.0001; Figure 1G), in sharp contrast to the reduced levels observed in males (−20.0%, *p* = 0.013; Figure 1H). TNF-α showed a genotype-specific elevation in females (+19.3%, *p* = 0.003; Figure 1I) with no genotype effect in males (*p* = 0.233; Figure 1J), although no significant sex × genotype interaction was detected (all *p* > 0.05; Supplementary Table S3).

Pathological analysis of moribund mice revealed a non-significant trend toward increased neoplasms in HKO-*Sirt6* mice (37.5% vs. 25.0% in Flox; Supplementary Figure S2B), with lesion complexity suggesting a non-lethal etiology. Assessment of DNA damage by quantifying γ-H2AX-positive foci (a marker of DNA double-strand breaks) revealed a robust sex × genotype interaction (*F*_(1, 10)_ = 15.74, *p* = 0.003; Supplementary Table S3). Aged HKO females demonstrated significantly increased γ-H_2_AX^+^ cells in centrilobular regions (*p* = 0.027 vs. Flox; Figure 1K), whereas males remained unaffected (young: *p* = 0.731; old: *p* = 0.814; Figure 1L). Despite increased DNA damage accumulation in females, no genotype- or sex-dependent differences in hepatocyte apoptosis or proliferation were observed (Supplementary Figure S3), indicating that *Sirt6* deficiency induces sex-specific DNA damage accumulation without immediate cell death.

Collectively, hepatocyte *Sirt6* deficiency drives female-preferential acceleration of aging, characterized by lifespan shortening, elevated IGF-1, pro-inflammatory dysregulation, and pronounced genomic instability.

### 2.2. Hepatocyte Sirt6 Deficiency Drives Sex-Specific Metabolic Aging Trajectories

**Figure 2:**
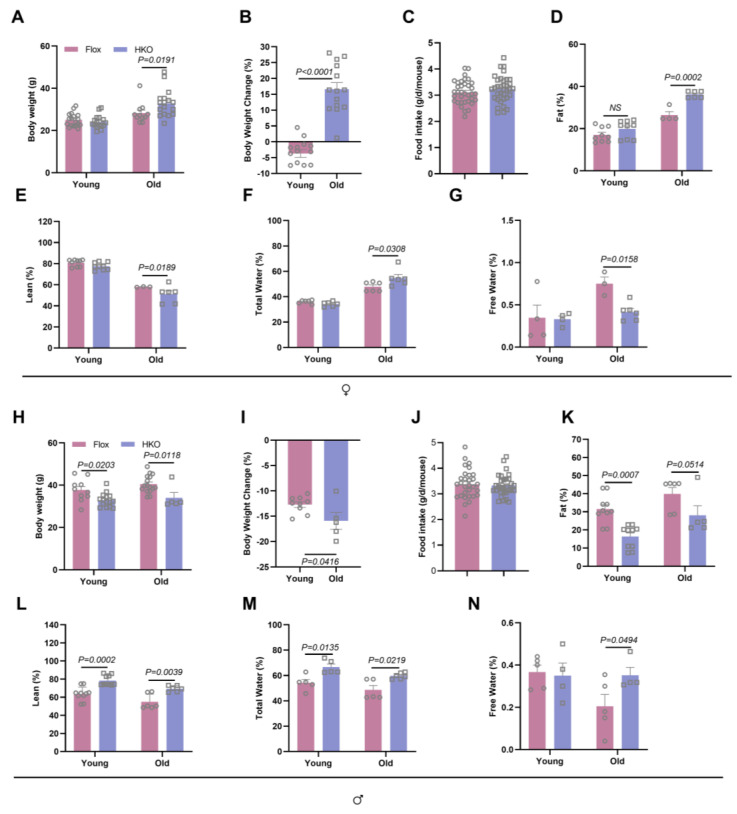
Hepatic *Sirt6* deficiency promotes age-related obesity exclusively in females. (**A**) Longitudinal body weight in female Flox and HKO mice at young (3–7 months) and aged (12–30 months) stages (*n* = 11–18 per group). (**B)** Percentage body weight gain in females relative to the baseline body weight of Flox littermates. (**C**) Average daily food intake (g/mouse/day) in females (*n* = 11–18 per group). (**D**–**G**) Body composition analyses by EchoMRI in females: Fat % (fat mass/body weight, (**D**)), Lean % (lean mass/body weight, (**E**)), Total water % (total water/body weight, (**F**)), and Free water % (free water/body weight, (**G**)) (*n* = 3–9 per group). (**H**–**N**) Parallel body composition analyses in male cohorts: Body weight (**H**), weight change percentage (**I**), food intake (**J**), and body composition parameters (**K**–**N**) (*n* = 4–10 per group). ♀: Female, ♂: Male. Data are presented as mean ± SEM. *p* values were determined by unpaired two-tailed *t*-test (**B**,**C**,**I**,**J**), and two-way ANOVA (age × genotype) with Dunnett’s (**A**,**D**,**E**,**H**,**K**,**L**), Tukey’s (**F**,**M**), or Fisher’s LSD (**G**,**N**) post hoc test. NS: not significant.

Aging is characteristically accompanied by progressive alterations in body composition, including the loss of lean mass and a redistribution of body fluids [42]. Our longitudinal body composition analysis revealed a profound sexual dimorphism governed by age-dependent sex × genotype interactions (Supplementary Table S3). In the aged cohort, female HKO-*Sirt6* mice exhibited a substantial 16.6% increase in body weight compared with their Flox littermates (*p* < 0.0001; Figure 2A,B), which occurred despite completely unchanged food intake (Figure 2C). This pathological weight gain was driven by a marked elevation in adiposity (+38.2% fat%, *p* = 0.0002; Figure 2D), a reciprocal decline in lean% (−16.7%, *p* = 0.0189; Figure 2E), and a disruption in fluid distribution characterized by increased total water % (*p* = 0.0308) and decreased free water % (*p* = 0.016; Figure 2F,G). Conversely, male HKO-*Sirt6* mice showed a progressive decline in body weight after 6 months of age, culminating in a 15.9% reduction at 16 months of age (*p* = 0.042; Figure 2H,I). This lean phenotype in males was associated with significantly reduced adiposity (−29.1% fat, *p* = 0.051 vs. Flox; Figure 2K), an increased lean% (*p* = 0.0039; Figure 2L), and an elevated fluid content in both total water % (*p* = 0.022 vs. Flox) and free water % (*p* = 0.0494 vs. Flox; Figure 2M,N), independent of any changes in food consumption (Figure 2J).

**Figure 3:**
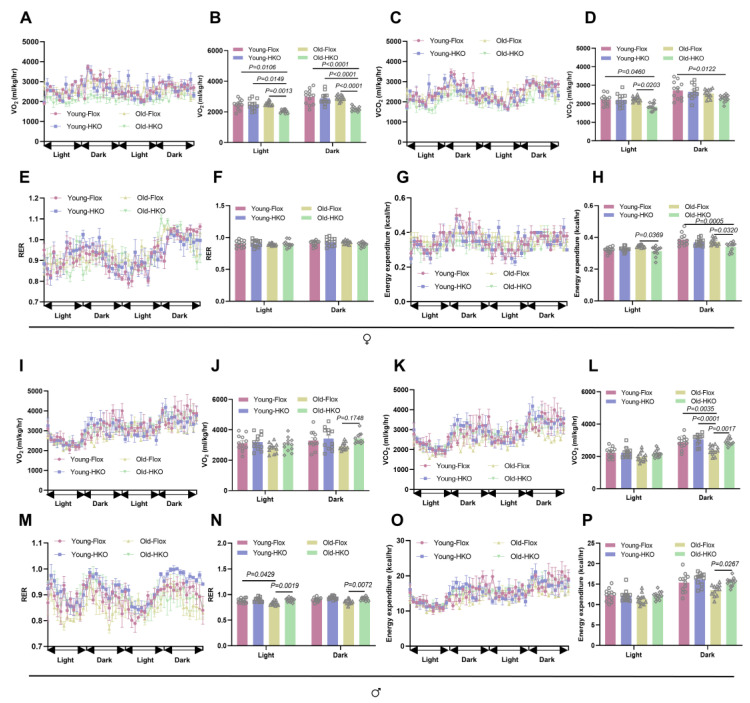
Hepatic *Sirt6* deficiency induces age-dependent metabolic impairment in females. Comprehensive metabolic phenotyping was performed using CLAMS (Columbus Instruments) in Flox and HKO mice during light/dark cycles (*n* = 3–4 independent mice per group). Female cohorts (**A**–**H**): (**A**,**B**) Oxygen consumption (VO_2_; mL/kg/h) in young and aged females. (**C**,**D**) Carbon dioxide production (VCO_2_; mL/kg/h). (**E**,**F**) Respiratory exchange ratio (RER = VCO_2_/VO_2_). (**G**,**H**) Energy expenditure. Male cohorts (**I**–**P**): Parallel analyses in young and aged males: VO_2_ (**I**,**J**), VCO_2_ (**K**,**L**), RER (**M**,**N**), and energy expenditure (**O**,**P**). ♀: Female, ♂: Male. All results are presented as mean ± SEM. *p* values were determined by a linear mixed-effects model with Tukey’s multiple comparisons test (**B**,**D**,**F**,**H**,**J**,**L**,**N**,**P**).

Comprehensive metabolic phenotyping uncovered the energetic basis underlying these divergent systemic trajectories. Two-way ANOVA identified significant sex × genotype interactions for fasting blood glucose (young: *p* = 0.0004; old: *p* = 0.0003), energy expenditure (old: *p* = 0.0002), and substrate utilization as assessed by the respiratory exchange ratio (RER = VCO_2_/VO_2_; old: *p* < 0.0001) (Supplementary Table S3). Specifically, aged female HKO-*Sirt6* mice developed a distinct hypometabolic phenotype. This was characterized by a significant reduction in oxygen consumption (VO_2_: −32.5% during the dark phase vs. Flox, *p* < 0.0001; Figure 3A,B), decreased CO_2_ production (VCO_2_: −18.5% during the light phase vs. Flox, *p* = 0.020; Figure 3C,D), and reduced total energy expenditure (−8.58% during the dark phase vs. Flox, *p* = 0.032; Figure 3G,H), while their RER remained unaltered (*p* > 0.05; Figure 3E,F). Interestingly, despite this suppressed metabolic rate and pronounced adiposity, female HKO-*Sirt6* mice maintained normal glucose homeostasis, as evidenced by unaltered fasting blood glucose levels, glucose tolerance (GTT), insulin sensitivity (ITT), and gluconeogenic capacity (PTT) (Supplementary Figure S4A–G).

In stark contrast, aged HKO male mice displayed a hypermetabolic and catabolic state. This was evidenced by significantly increased O_2_ consumption (+17.6% during the dark phase, Figure 3I,J), elevated CO_2_ production (+21.5% during the dark phase, *p* = 0.002; Figure 3K,L), and higher systemic energy expenditure (+14.8% during the dark phase, *p* = 0.027; Figure 3O,P). Furthermore, males exhibited a significantly raised dark-phase RER (shifting from 0.87 to 0.98 dark phase, *p* = 0.0072; Figure 3M,N), indicating a preferential shift toward carbohydrate oxidation. This active metabolic acceleration in males was accompanied by markedly reduced fasting glucose levels in both young (−30.8%, *p* = 0.025) and old (−43.5%, *p* < 0.0001 vs. Flox; Supplementary Figure S4H) cohorts, although their insulin sensitivity and gluconeogenesis showed non-significant trends (Supplementary Figure S4I–N).

Collectively, these data demonstrate that hepatocyte-specific *Sirt6* deletion induces sex-divergent metabolic aging trajectories. Females transition into a hypometabolic, energy-conserving state characterized by progressive adiposity and lean mass loss, whereas males activate a resilient catabolic state associated with improved glycemia and adiposity.

### 2.3. Hepatocyte Sirt6 Deficiency Drives Sex-Divergent Progression of Metabolic-Associated Fatty Liver Disease

**Figure 4:**
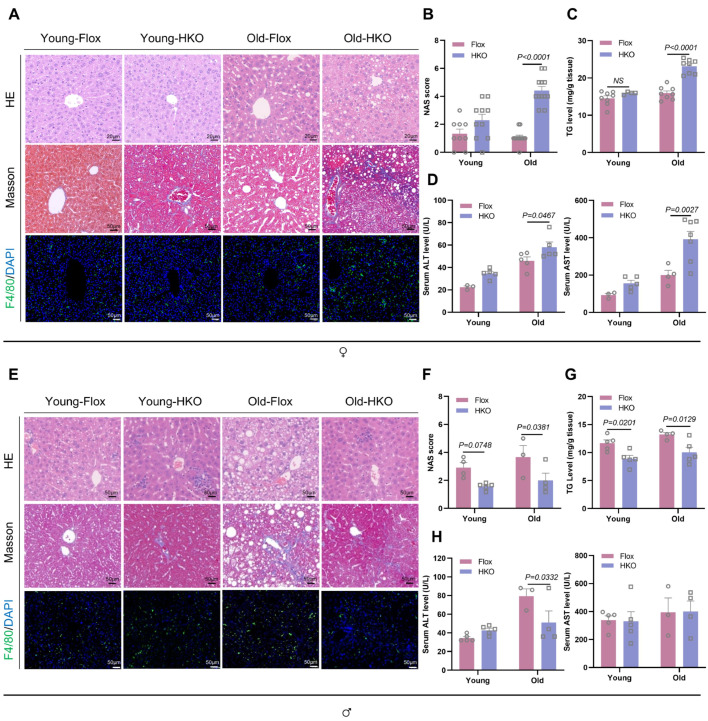
Hepatic *Sirt6* deficiency drives age-dependent fatty liver in female mice, but not in males. (**A**,**E**) Representative liver histological images from female (**A**) and male (**E**) mice: H&E staining (top), Masson’s trichrome staining (middle), and immunofluorescence for F4/80^+^ macrophages (green) with DAPI nuclear counterstain (bottom) in liver sections. Scale bar, 20/50 μm (*n* = 3–6 biologically independent mice per group). (**B**,**F**) Non-alcoholic fatty liver disease (NAFLD) activity score (NAS) quantification including steatosis (0–3), lobular inflammation (0–3), and ballooning (0–2) subscores (*n* = 3–5 per group). (**C**,**G**) Hepatic triglyceride (TG) concentrations in female (**C**) and male (**G**) mice (*n* = 4–8 per group). (**D**,**H**) Serum levels of ALT and AST in females (**D**) and males (**H**) (*n* = 3–7 per group). ♀: Female, ♂: Male. Data are presented as mean ± SEM. *p* values were determined by two-way ANOVA with Sidak’s multiple comparisons test (**B**–**D**,**F**–**H**).

**Figure 5:**
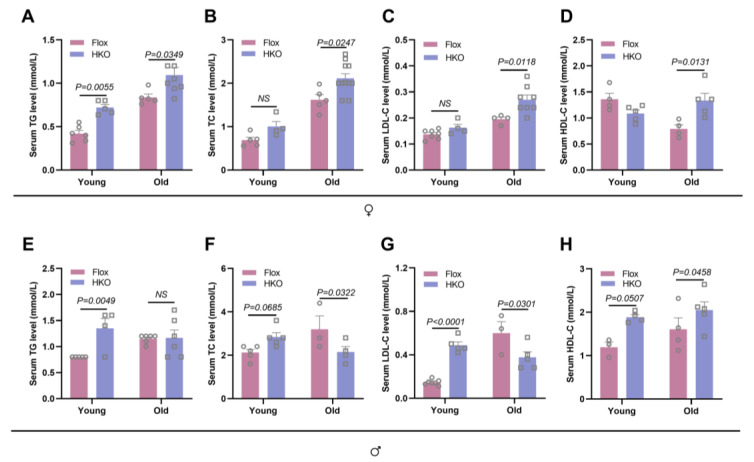
Liver-specific *Sirt6* deficiency causes female-specific age-dependent dyslipidemia. Fasting serum lipid profiles were measured in both young and aged Flox and HKO mice. Female cohorts (**A**–**D**): serum triglycerides (TG, **A**), total cholesterol (TC, **B**), low-density lipoprotein cholesterol (LDL-C, **C**), and high-density lipoprotein cholesterol (HDL-C, **D**) levels in female mice (*n* = 4–11 per group). Male cohorts (**E**–**H**): Serum TG (**E**), TC (**F**), LDL-C (**G**)**,** and HDL-C (**H**) levels in male mice (*n* = 3–6 per group). ♀: Female, ♂: Male. Data are presented as mean ± SEM. *p* values were determined by two-way ANOVA with Sidak’s post hoc test (**A**,**G**), Fisher’s LSD test (**F**), or Tukey’s post hoc analysis (**B**–**D**,**E**,**H**).

To determine the impact of hepatic SIRT6 on lipid homeostasis, we performed a longitudinal analysis of liver histopathological profiles and serum biochemistry across age cohorts. Comprehensive analysis revealed profound sex × genotype interactions in hepatic pathology during the aging process (Supplementary Table S3). Female HKO-*Sirt6* mice developed progressive hepatic steatosis beginning as early as 4 months of age. Strikingly, aged females exhibited an accelerated progression of MAFLD, with 41.7% (5 out of 12) fulfilling the histological criteria for early-stage metabolic-associated steatohepatitis (MASH), defined by a non-alcoholic fatty liver disease (NAFLD) activity score (NAS) ≥ 4. Histopathological evaluation demonstrated significant microvesicular and macrovesicular steatosis, portal inflammation accompanied by F4/80^+^ macrophage infiltration, and perisinusoidal fibrosis in HKO-*Sirt6* in aged female HKO livers (Figure 4A). These structural impairments were firmly supported by biochemical analyses, which demonstrated marked lipid accumulation and liver damage, including elevated hepatic triglycerides (TG: +45.3%, *p* < 0.0001 vs. Flox; Figure 4C) and increased classical indicators of hepatocellular injury, serum alanine aminotransferase (ALT: +26.1%, *p* = 0.047; Figure 4D) and aspartate aminotransferase (AST: +95.3%, *p* = 0.003; Figure 4D). This localized hepatic collapse was mirrored systematically by progressive dyslipidemia, manifested as increased serum levels of TG (+ 30.8%, *p* = 0.035; Figure 5A), total cholesterol (TC: +30.3%, *p* = 0.025; Figure 5B), LDL-C (+38.5%, *p* = 0.012; Figure 5C), and HDL-C (+69.1%, *p* = 0.0131; Figure 5D).

Conversely, aged male HKO-*Sirt6* mice exhibited remarkable protection against age-related hepatic steatosis, displaying an attenuated pathological trajectory. Compared with their aged-matched Flox controls, aged HKO-*Sirt6* males showed an alleviated NAS (−42.9%, *p* = 0.038; Figure 4E,F), a substantial decrease in hepatic TG accumulation (−24.0%, *p* = 0.013; Figure 4G), and markedly reduced serum ALT levels (−35.7%, *p* = 0.033; Figure 4H), which correlated with minimal fibrotic deposition and macrophage infiltration. Furthermore, the serum lipid profiles in male mice underwent an age-dependent normalization (Figure 5E–H). This systemic lipid-clearing phenotype perfectly aligned with their enhanced catabolic state (characterized by increased energy expenditure and a shift toward carbohydrate utilization) and improved fasting glucose (−43.5%, *p* < 0.0001).

Collectively, these data demonstrate that hepatocyte-specific *Sirt6* deficiency produces a female-preferential progression of MAFLD through age-amplified lipotoxicity, chronic inflammation, and parenchymal injury. In contrast, the loss of hepatic SIRT6 paradoxically protecting aged males from MAFLD via a resilient metabolic reprogramming.

### 2.4. Hepatic Sirt6 Deficiency Drives SULT1E1-Mediated Transcriptional Reprogramming, Whereas Pharmacological SIRT6 Activation Prevents Female-Biased Pathological Aging

**Figure 6:**
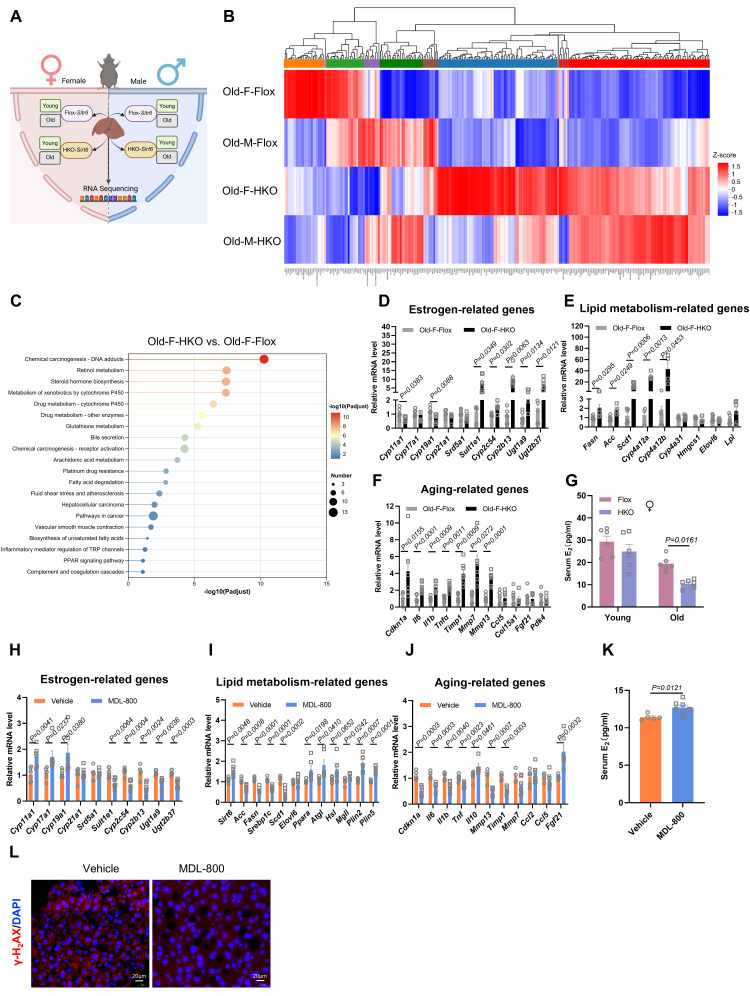
Hepatic *Sirt6* deficiency drives female-biased estrogen-metabolic transcriptomic reprogramming, whereas SIRT6 activator MDL-800 rescues aging via *Sult1e1* suppression. (**A**) Experimental design for RNA sequencing on livers from young (6-month) and old (18-month) Flox-*Sirt6* and HKO-*Sirt6* mice (*n* = 3 biologically independent mice per group per sex). (**B**) Hierarchical clustering heatmap representing differentially expressed genes (DEGs; |log_2_FC| ≥ 1, adjusted *p* < 0.05) in aged cohorts. Relative expression levels are displayed as Z-score normalized values (blue: downregulated, red: upregulated). (**C**) Top enriched KEGG pathways of DEGs in aged HKO females ranked by −log_10_ (adjusted *p* value). (**D**–**G**) Female-specific molecular pathology in aged HKO mice: qPCR validation of hepatic estrogen catabolic genes (**D**), dysregulated lipid metabolism genes (**E**), and aging-associated genes (**F**), systemic serum 17β-estradiol (E_2_) levels quantified by ELISA (**G**) (*n* = 6 per group). (**H**–**K**) Therapeutic effects of the pharmacological SIRT6 activator MDL-800 in aged females: Relative hepatic expression of estrogen signaling and metabolic genes (**H**), lipogenesis and lipolysis-related genes (**I**), and senescence-associated marker and SASP genes (**J**) following MDL-800 treatment (100 mg/kg/day) (*n* = 4–6 per group). (**K**) The levels of E_2_ of mice exposed to vehicle and MDL-800 (*n* = 5–7 per group). (**L**) Representative immunofluorescence images of γ-H_2_AX^+^ foci (red) counterstained with DAPI (blue) in the liver (*n* = 4–5 per group). Scale bar, 20 μm. ♀: Female, ♂: Male. Data in (**D**–**J**) are presented as mean ± SEM. *p* values were determined by multiple *t*-test (**D**–**F**,**H**–**J**), two-way ANOVA with Sidak’s multiple comparisons test (**G**) and unpaired two-tailed *t*-test (**K**).

To elucidate the molecular basis for sex-divergent aging, we performed transcriptomic profiling via RNA sequencing on liver tissues from Flox- and HKO-*Sirt6* mice across both sex and age cohorts (Figure 6A). In aged mice, HKO-*Sirt6* deletion triggered sex-specific pathological transcriptomic remodeling. Aged HKO female livers exhibited 254 differentially expressed genes (DEGs: 197 upregulated, 57 downregulated; log_2_|FC| ≥ 1, adjusted *p* < 0.05). KEGG pathway enrichment analysis of these DEGs showed a female-exclusive activation of pathways associated with DNA adduct formation (*p* = 5.15 × 10^−11^), steroid hormone biosynthesis (*p* = 3.86 × 10^−8^), and xenobiotic metabolism (*p* = 3.93 × 10^−8^) (Figure 6B,C and Supplementary Figure S5A,B). These pathways were also prominently enriched when directly comparing aged HKO females and their male counterparts (Supplementary Figure S5C).

In stark contrast, aged HKO male mice displayed a profoundly different protective transcriptional landscape (236 upregulated and 249 downregulated DEGs; Figure 6B and Supplementary Figure S5A,B). Notably, males exhibited a highly selective enrichment in thyroid hormone synthesis (*p* = 0.003) and glutathione metabolism (*p* = 0.041; Supplementary Figure S5D), alongside adaptive metabolic shifts without senescence activation in young HKO cohorts (Supplementary Figure S5E,F). This suggests that male mice launch active, compensatory metabolic networks to maintain hepatic homeostasis.

Given the liver’s central role in systemic endocrine regulation, we hypothesize hepatic SIRT6 governs aging trajectories primarily through the control of steroid hormone metabolism. A direct comparison between aged HKO females and males confirmed a SULT1E1-centered sexual dimorphism in steroid metabolism. qPCR validation identified three interconnected pathological axes operating exclusively in aged female livers: (1) Estrogen catabolism dominance: a dramatic upregulation of estrogen-inactivating enzymes, including *Sult1e1* (+7.54-fold, *p* = 0.035), *Cyp2b13* (+10.5-fold, *p* = 0.006) and *Ugt2b37* (+7.11-fold, *p* = 0.012), accompanied by the suppression of key steroidogenic genes such as *Cyp19a1* (−0.720-fold) and *Cyp11a1* (−0.669-fold) (Figure 6D); (2) Accelerated lipogenesis: severe lipogenic activation marked by elevated expression of *Cyp4a12a* (+30.2-fold), *Cyp4a12b* (+42.8-fold), *Scd1* (+15.2-fold), *Fasn* (+2.03-fold), and *Acc* (+1.78-fold) (*p* < 0.05 vs. Flox; Figure 6E); (3) Senescence and SASP induction: a robust induction of cellular senescence and the senescent-associated secretory phenotype (SASP), characterized by upregulation of *Mmp7* (+5.09-fold), *Cdkn1a* (p21, +4.24-fold), *Il6* (+2.27-fold), *Il1b* (+2.36-fold), *Tnf* (+2.36-fold), *Timp1* (+3.97-fold), and *Mmp13* (+3.47-fold) (*p* < 0.05 vs. Flox; Figure 6F).

This multi-axial dysregulation was further highlighted by a direct comparison between aged HKO females and males, revealing a marked female-specific amplification of altered steroid metabolism (*Sult1e1*: +5.41-fold, *Cyp11a1*: −0.433-fold, *Cyp19a1*: −0.577-fold), lipogenesis (*Acc*: +2.58-fold, *Fasn*: +2.90-fold, *Elovl6*: +3.43-fold), and cellular senescence (*p21*: +4.92-fold, *Ccl5*: +2.41-fold) (Supplementary Figure S5G–I). Crucially, this localized hepatic shift in estrogen catabolism culminated in a 46.10% reduction in systemic circulating estradiol (E_2_) levels in female mice (*p* = 0.016; Figure 6G), while circulating testosterone levels in males remained unchanged (Supplementary Figure S5J). As the liver functions as the primary hub for steroid metabolism, the aberrant induction of SULT1E1 in HKO female livers establishes a potent systemic “estrogen sink”, depleting the protective active estradiol necessary for systemic metabolic health. Mechanistically, this triggers a pathological loop: SULT1E1-mediated estrogen inactivation via sulfonation promotes lipid accumulation and oxidative stress, while concomitant cytochrome P450 dysregulation (e.g., Cyp4a12a/Cyp2c54) increases ROS production to accelerate genomic damage. Coupled with the impaired DNA repair caused by *Sirt6* deficiency, this cascade leads to rapid DNA adduct accumulation, genomic instability and widespread cellular senescence, exclusively in female mice.

To validate the translational potential of these findings, we treated aged female mice with MDL-800, a potent and specific SIRT6 activator developed by our group [39]. Remarkably, pharmacological SIRT6 activation by MDL-800 successfully rescued the female biased pathological aging phenotypes. MDL-800 significantly restored hepatic SIRT6 expression (+ 44.2%, *p* = 0.005), while concurrently suppressing *Sult1e1* expression (−34.8%, *p* = 0.006). MDL-800 treatment increased circulating serum E_2_ levels compared with vehicle-treated mice (+11.30%, *p* = 0.0121) (Figure 6K). This was accompanied by the downregulation of key phase I/II drug-metabolizing enzymes (*Cyp2c54*: −0.648-fold; *Ugt2b37*: −0.694-fold) and upregulation of steroidogenic genes *Cyp11a1* (1.73-fold) and *Cyp19a1* (1.86-fold), reflecting a restoration of local estrogen homeostasis. Furthermore, MDL-800 treatment reinstated metabolic plasticity by downregulating de novo lipogenesis (Scd1: −58.6%) and upregulating lipolysis (*Plin2*: +78.0% *Ppara*: +42.3%). It also attenuated cellular senescence (*p21*: −0.497-fold; *Fgf21*: +2.033-fold), dampened the pro-inflammatory SASP signature (*Tnf*: −35.8%; *Il10*: +31.6%) (Figure 6H–J), and significantly reduced γ-H_2_AX foci formation without altering baseline rates of apoptosis or proliferation (Figure 6L and Supplementary Figure S6).

In summary, these data demonstrate that hepatocyte-specific *Sirt6* deficiency drives female-specific systemic aging and metabolic decline through SULT1E1-dependent estrogen depletion, whereas pharmacological activation of SIRT6 with MDL-800 provides a viable therapeutic strategy to reverse these gerometabolic phenotypes.

## 3. Discussion

Our study establishes hepatic SIRT6 as a critical modulator of sexually dimorphic aging, where pervasive sex × genotype interactions drive divergent pathological outcomes. Robust statistical interactions across multiple phenotypes, including aging-related markers (circulating IGF-1, pro-inflammatory cytokines, and hepatic γH_2_AX foci), metabolic parameters (VO_2_, energy expenditure), and MAFLD progression (NAS, liver TG, and serum transaminases), confirm that hepatic *Sirt6*-dependent pathways operate through fundamentally sex-divergent mechanisms. Female HKO-*Sirt6* mice exhibited an acceleration of natural aging, manifested by a 17.60% reduction in median lifespan. This premature senescence was phenotypically characterized by gerometabolic deterioration (including visceral obesity, advanced MAFLD, and systemic dyslipidemia) and cellular senescence, marked by genomic instability (γH_2_AX foci accumulation; *F*_(1, 10)_ = 15.74, *p* = 0.003) and the upregulation of canonical senescence biomarkers (p21, TNF-α, and IGF-1). Strikingly, HKO-*Sirt6* males maintained metabolic homeostasis, displaying improved adiposity and hepatic protection. This highlights how these statistically validated interactions translate to diametrically opposing phenotypic outcomes between sexes. This female-biased vulnerability stands in stark contrast to typical mammalian longevity patterns and underscores the liver’s pivotal role in orchestrating sex-divergent aging trajectories.

Crucially, our findings address a fundamental question regarding the systemic impact of a cell-type-specific deletion, demonstrating that SIRT6 expression in female hepatocytes regulates lifespan and healthspan phenotypes in a major, organismal-level manner. This long-range control is centrally driven by estrogen sulfotransferase (SULT1E1), which catalyzes the sulfonation of active estrogens (e.g., 17β-estradiol, E_2_) into biologically inert sulfates incapable of binding ERα [43]. In female HKO-*Sirt6* mice, the transcriptional hyperactivation of hepatic *Sult1e1* (+7.54-fold) triggered a systemic hormonal collapse, evidenced by a 46.10% decrease in circulating E_2_ levels. Because the liver serves as the primary metabolic and clearance hub for systemic steroid hormones [44], this aberrant hepatic upregulation establishes a female-specific systemic “estrogen sink” mechanism. This resulting depletion of circulating E_2_ effectively strips extrahepatic tissues, such as adipose tissues, skeletal muscle, and the cardiovascular system [45,46], of estrogen-mediated anti-inflammatory and antioxidant protections [47]. This systemic hormonal deprivation explains why a localized hepatocyte deficiency culminates in widespread, organism-level physiological decline. Notably, while absolute circulating E_2_ levels in aged female rodents reflect recognized low baseline ranges reported in rodent reproductive senescence, the significant drop in HKO females directly reflects hepatic E_2_ depletion. Importantly, pharmacological SIRT6 activation by MDL-800 successfully restored circulating E_2_ availability, confirming that targeting SIRT6 effectively dismantles this estrogen sink. This mechanism is strongly confirmed by established literature: (1) Hepatic *Sult1e1* ablation improves insulin sensitivity exclusively in females while exacerbating metabolic stress in males [48]; (2) *Sult1e1* displays strict tissue- and sex-specific expression profiles, being predominantly hepatic in females but restricted to white adipose tissue (WAT) in males under testosterone-dependent regulation [49,50]; (3) Adipose *Sult1e1* reconstitution rescues metabolic function in obese male mice but fails to do so in females [51].

Although SULT1E1 has been reported to promote MAFLD progression in mixed-sex cohorts [52,53], its aberrant induction following hepatic *Sirt6* deficiency uniquely perturbs female-specific estrogen signaling, initiating a self-amplifying pathological triad. First, active estrogen depletion derepresses master lipogenic gene expression (such as *Scd1*/*Fasn*) to drive hepatic steatosis. Second, concomitant CYP450 dysregulation (e.g., upregulation of *Cyp2c54* and *Cyp2b13*) facilitates the formation of reactive metabolites and DNA adducts, which triggers a compensatory yet insufficient upregulation of GST detoxification (*Gstm/Gsta*), ultimately exacerbating genomic instability [54,55]. Third, the loss of SIRT6-impaired DNA repair further accelerates cellular senescence and fuels the pro-inflammatory SASP cascade (upregulation of *p21*/*IL-6*) [56]. At the transcriptional level, SULT1E1 is tightly governed by a network of nuclear receptors, including direct repression by FXR through the inhibition of PGC1α-HNF4α interactions [57], and induction by LXR activation [58]. Notably, these nuclear receptors are direct targets of SIRT6-dependent epigenetic and post-translational remodeling: SIRT6 stabilizes ERα via lysine171 and 299 deacetylation to preserve estrogen sensitivity [30], enhances FXR transcriptional activity through deacetylation [59], and suppresses the pro-lipogenic LXR/SREBP-1c pathway by directly deacetylating LXR [60,61]. This positions SIRT6 as a master coordinator of nuclear receptor crosstalk, maintaining estrogen homeostasis and protecting against female-specific accelerated aging.

In sharp contrast to the catastrophic hormonal and metabolic collapse observed in females, male HKO-*Sirt6* mice exhibited remarkable metabolic resilience, uncovering a distinct, male-specific compensatory framework. Because baseline circulating E_2_ levels are negligible in males, they do not rely on the hepatic estrogen-ERα axis for metabolic preservation. More importantly, our comprehensive transcriptomic profiling revealed that male mice actively launch robust compensatory networks upon hepatic SIRT6 loss to maintain cellular and systemic homeostasis. Specifically, aged HKO male livers displayed a highly selective enrichment in thyroid hormone synthesis and glutathione metabolism pathways, alongside adaptive lipid metabolism networks (such as PPARα targets). This active metabolic reprogramming effectively counteracts lipotoxicity, maintains antioxidant capacity, and prevents the activation of cellular senescence in males. These context- and sex-dependent actions of SIRT6 suggest that while systemic SIRT6 activation enhances genome stability and suppresses inflammation across both sexes [62,63], its tissue-specific therapeutic activation requires caution, as it may differentially impinge upon IGF-1, hormone, and redox pathways in male versus female [64,65].

These statistically grounded sex differences support stratified therapeutic strategies targeting the SIRT6 – SULT1E1 axis. In females, SIRT6 activation via MDL-800 (a small-molecule activator developed by our group) [39] or potential SULT1E1 inhibition (e.g., flavonoid-based inhibitors like quercetin) restores active estrogen signaling, alleviates gerometabolic vulnerability [66], and reduces genotoxic estrogen-derived metabolites implicated in tumor initiation [67]. Mechanistically, female-specific vulnerability is driven by SIRT6-mediated control of estrogen signaling through deacetylation of ERα and ERRγ [30,68], whereas male metabolic resilience operates through estrogen-independent networks. MDL-800-mediated SIRT6 activation comprehensively rescues the female-specific aging phenotype by suppressing *Sult1e1* suppression (↓34.8%), restoring circulating E_2_ availability (*p* = 0.0121), enhancing genomic stability, and restoring metabolic flexibility. Furthermore, organismal longevity is coordinated by a multi-mediator heaptic network. While baseline hepatic *Fgf21* expression remained unchanged in HKO females, SIRT6 activation by MDL-800 significantly induced *Fgf21* expression (+2.03-fold, *p* = 0.0032), demonstrating that SIRT6 confers synergistic protection through both estrogen-sink dismantling and hepatokine-mediated metabolic rejuvenation. Future studies exploring additional liver-derived metabolites, such as taurine, will further define this integrated network. Our study defines a druggable sexual dimorphism in aging interventions. These distinct regulatory frameworks underscore the necessity of sex-matched evaluation of longevity therapeutics and raise the possibility of combining SIRT6 activators with SULT1E1 inhibitors to achieve therapeutic synergy in females.

### Significance and Limitations

Our study identifies hepatic SIRT6-SULT1E1 axis governing sex-divergent aging trajectories, supported by rigorous statistical evidence of sex × genotype interactions across multiple aging phenotypes. In conclusion, we uncover three key advances: (1) A unifying mechanism: Hepatocyte SIRT6 ablation triggers SULT1E1 hyperactivation exclusively in females, initiating a self-amplifying cascade of steroid hormone disruption, lipotoxicity and DNA damage that underlies female-specific metabolic collapse and tumor susceptibility; (2) A sex-stratified therapeutic paradigm: Small-molecule activation of SIRT6 via MDL-800 restores estrogen homeostasis and mitigates multiple aging-related pathologies specifically in females, while males adapt through alternative estrogen-independent pathways; (3) A conceptual shift: Our findings emphasize the necessity of incorporating sex-matched models in aging research, as tissue-specific sirtuins actions can yield profoundly divergent phenotypes depending on hormones. However, several limitations should be considered. First, the use of a constitutive hepatocyte-specific *Sirt6* knockout may allow developmental compensatory mechanisms. Second, our study focused exclusively on the liver. Whether SIRT6 exerts similar sex-specific roles in other major metabolic tissues, such as adipose tissue and skeletal muscle, remains unknown. Finally, the long-term efficacy of MDL-800 on actual lifespan extension requires validation in separate, dedicated cohorts, and its translational relevance awaits confirmation using human clinical samples.

## 4. Materials and Methods

### 4.1. Mice Study

All mice were housed in individually ventilated cages in a specific pathogen-free (SPF) facility under controlled conditions, including temperature (23 ± 2 °C), relative humidity (50 ± 10%) and 12-h light/dark cycles. Mice had free access to food and water except during designated fasting periods. All procedures were conducted in accordance with the guidelines approved by the Institutional Animal Care and Use Committee of Shanghai Jiao Tong University School of Medicine (Approval No.: JUMC2023-109-A and date of approval: 9 October 2024), with strict adherence to the ethical principles aimed at minimizing animal suffering and reducing the number of animals used.

*Sirt6*^flox/flox^ mice carrying loxP sites flanking exons 2–3 of the *Sirt6* gene were obtained from The Jackson Laboratory (Cat# 017334, The Jackson Laboratory, Bar Harbor, ME, USA). This strain was originally generated using 129S6/SvEvTac-derived embryonic stem cells and subsequently backcrossed onto an FVB/NJ background by the donating investigator. The mice were backcrossed onto the pure C57BL/6J background for at least 10 generations in our facility prior to experimental intercrossing with Alb-Cre transgenic mice. Hepatocyte-specific *Sirt6* knockout mice (HKO-*Sirt6*; genotype: *Sirt6*^flox/flox^; *Alb-Cre^+^*) were generated by crossing *Sirt6*^flox/flox^ mice with albumin-Cre transgenic mice [37]. Homozygous littermate controls were *Sirt6*^flox/flox^; Alb-Cre (Flox-*Sirt6*). After weaning, mice were sex-separated, ear-tagged, and genotyped using DNA extracted from toe or tail biopsies. Genotyping primers were as follows: Flox-*Sirt6* (5′-GCTAATGGGAACGAGACCAA-3′; 5′-ACCCACCTCTCTCTCCCTAAA-3′; WT: 390 bp, Flox: 444 bp); Alb-Cre (5′-GCGGTCTGGCAGTAAAAACTATC-3′; 5′-AGCAATCCCCAGAAATGCCAG-3′; Cre^+^ 260 bp). Age stratification: young adults: 3–7 months; aged cohorts: 12–30 months.

To evaluate the effects of the pharmacological SIRT6 activator on aging, 6-month-old female Flox-mice were fed a high-fat diet (HFD, 60 kcal% fat, 20 kcal% protein, 20 kcal% carbohydrates; Cat# D12492, Research Diets, New Brunswick, NJ, USA). After 24 weeks of HFD feeding, mice were randomly assigned to two groups and received HFD supplemented with either vehicle or MDL-800 (100 mg/kg/day) for 8 weeks.

### 4.2. Collection of Tissue and Blood Samples

Following a 12-h overnight fast, mice were anesthetized by intraperitoneal injection of Zoletil^®^50 (Tiletamine and zolazepam, 5.00–7.50 mg/kg for mice). Terminal blood collection was performed by cardiac puncture. Blood was clotted for 1 h at room temperature (RT), and then centrifuged at 4500× *g* for 10 min to obtain serum. Serum aliquots were stored at −80 °C for subsequent analyses. Tissues were dissected, weighed and processed as follows: The left lateral liver lobe was fixed in 4% paraformaldehyde (PFA) for 12 h at RT before paraffin embedding. The median liver lobe was snap-frozen in liquid N_2_ for RNA and protein extraction. The right liver lobes were freshly frozen in OCT for cryosectioning. Other tissues, such as white adipose tissue (WAT), skeletal muscle, brain, kidney and brown adipose tissue, were rapidly frozen in liquid nitrogen and stored at −80 °C for subsequent analyses.

### 4.3. Lifespan Assessment

For lifespan assessment, cohorts of mice were housed under standard conditions with minimal disturbance and monitored daily for health status until natural death. Survival events were recorded systematically. Mice exhibiting severe frailty (defined as meeting ≥ 3 of the following criteria: severe weight loss > 20%, impaired mobility, hunched posture, or ulcerative tumors) were humanely euthanized via CO_2_ asphyxiation and included in survival analysis [25]. Genotyping was performed only at weaning to minimize disturbance. Survival curves were generated using Kaplan–Meier analysis in GraphPad Prism 9 (version 9.5.1). Statistical significance of *p* values was determined by the log-rank (Mantel–Cox) test and the Gehan-Breslow-Wilcoxon test comparing sex-matched HKO-*Sirt6* and Flox-*Sirt6* groups. Maximum lifespan was defined as the age of the last surviving mouse in each group.

### 4.4. Serum Hormone and Glucose Metabolism Analyses

Blood samples were collected from the orbital venous plexus under brief isoflurane anesthesia following a 16-h overnight fast. For hormone testing, blood collection was performed at a fixed time window (9:00 A.M.–10:00 A.M.) after overnight fasting. Serum was obtained after allowing blood samples to clot for 1 h at room temperature, followed by centrifugation at 4500× *g* for 10 min, and was then stored at −80 °C for batch analysis. Circulating IGF-1 (Proteintech, Wuhan, China, Cat# KE10032), estradiol (E_2_) (Cat# MEA461Ge, Cloud-Clone Corp, Wuhan, China), testosterone (Cat# MEA458Ge, Cloud-Clone Corp, Wuhan, China), IL-6 (Cat# SM6000B, R&D Systems, Minneapolis, MN, USA) and TNF-α (Cat# SMTA00B, R&D Systems, Minneapolis, MN, USA) levels were quantified using ELISA kits, according to the manufacturer’s instructions.

Glucose metabolism was evaluated through sequential tolerance tests. For the glucose tolerance test (GTT), overnight-fasted (16 h) mice of the indicated age and sex received glucose (2 g/kg body weight) via intragastric gavage. For the pyruvate tolerance test (PTT), sodium pyruvate (1.00 g/kg body weight) was injected intraperitoneally into mice after 16-h fasting. For the insulin tolerance test (ITT), mice were intraperitoneally injected with insulin (0.75 IU/kg) following 4-h fast (9:00 A.M.–2:00 P.M.). Blood glucose levels were measured from tail vein blood at 0, 15, 30, 60, 90, and 120 min after administration using a BAYER Contour Plus glucometer.

### 4.5. Body Composition and Metabolic Function Assessments

Body composition was assessed in conscious mice using the EchoMRI-100 analyzer (Echo Medical Systems, Houston, TX, USA). Fat mass, lean mass, free water, and total water measurements were normalized to individual body weight. Whole-body energy metabolism was assessed using a Columbus Instruments Comprehensive Lab Animal Monitoring System (CLAMS; Columbus Instruments, Columbus, OH, USA) metabolic cages. After 48-h acclimation, mice were continuously monitored for 72 h. Oxygen consumption (VO_2_), carbon dioxide production (VCO_2_), respiratory exchange ratio (RER = VCO_2_/VO_2_), and energy expenditure were recorded at approximately 10-min intervals under standardized light/dark cycles.

### 4.6. RNA Extraction and Quantitative Real-Time PCR (qPCR)

Total RNA was extracted from liver tissues using the RNA extraction kit (Cat# RC112, Vazyme, Nanjing, China) following the manufacturer’s instructions. RNA concentration and purity were spectrophotometrically assessed (A260/A280). cDNA was synthesized from 1 μg of total RNA using the HiScriptIII RT supermix (Cat# R323, Vazyme, Nanjing, China). qPCR was performed in triplicate using ChamQ Universal SYBR qPCR Master Mix (Cat# Q711, Vazyme) on a QuantStudio 1 system (Applied Biosystems, Thermo Fisher Scientific, Carlsbad, CA, USA). The amplification conditions were 95 °C for 15 s, followed by 40 cycles of 95 °C for 15 s and 60 °C for 1 min. Cycle threshold (Ct) values were normalized to *Gapdh* using the 2^−ΔΔCt^ method. Primer sequences are listed in Supplementary Table S1.

### 4.7. Western Blotting

Liver tissues were homogenized in RIPA lysis buffer (Cat# P0013B, Beyotime, Shanghai, China) supplemented with 1 mM dithiothreitol, 1× protease/phosphatase inhibitor cocktail (Cat# 78842, Thermo Fisher Scientific, USA). Lysates were centrifuged at 12,000× *g* for 15 min at 4 °C, and supernatants were collected. Protein concentrations were determined using the BCA protein assay kit (Cat# ZJ101, Epizyme, Shanghai, China). Equal amounts of protein were separated by 10% SDS-PAGE and transferred to PVDF membranes (Millipore, Burlington, MA, USA). Membranes were blocked with 5% non-fat milk for 1 h at RT and incubated overnight at 4 °C with primary antibodies against SIRT6 (1:1000, Cat# 12486S, Cell Signaling Technology, Boston, MA, USA) or β-actin (1:2000, Cat# HRP-60008, Proteintech, Wuhan, China). Protein bands were visualized using ECL substrate on an Amersham ImageQuant 800 system (Cytiva, Chicago, IL, USA).

### 4.8. Histological Analysis, Immunohistochemistry and Immunofluorescence

Liver tissues were fixed in 4% paraformaldehyde, paraffin-embedded, and sectioned at a thickness of 4 μm. Sections were stained with hematoxylin and eosin (H&E) to evaluate hepatic steatosis, inflammation, and hepatocyte ballooning, or subjected to Masson’s trichrome staining to assess collagen deposition. For immunohistochemistry, paraffin sections were deparaffinized, rehydrated, and subjected to antigen retrieval. Endogenous peroxidase activity was quenched with 3% hydrogen peroxide for 30 min. Liver sections were then incubated with antibodies against γ-H_2_AX (1:100, Cat# GB111841, Servicebio, Wuhan, China), TUNEL (1:50, Cat# G1507, Servicebio, Wuhan, China) and Ki-67 (1:800, Cat# GB151499, Servicebio, Wuhan, China). Slides were scanned using a Pannoramic 250FLASH slide scanner (3DHISTECH Ltd., Budapest, Hungary) controlled by CaseViewer software (version 2.4; 3DHISTECH Ltd., Budapest, Hungary). The MAFLD activity score (NAS) was blindly assessed to evaluate liver steatosis (0–3), lobular inflammation (0–3), hepatocellular ballooning (0–2), and fibrosis (0–4) [69]. The AIpathwell software (version 2.0; Servicebio Technology Co., Ltd., Wuhan, China) was used to analyze the positive staining according to the manufacturer’s instructions. γ-H_2_AX expression was quantified using the H-score method, which provides a comprehensive assessment of both the percentage of positively stained cells (Pi) and their staining intensity (i) according to the following formula:

H-score = Σ (Pi × i)

where i represents staining intensity (0: negative; 1+: weak; 2+: moderate; 3+: strong). The theoretical score range is 0–300, with higher values indicating stronger immunoreactivity [70]. For immunofluorescence, paraffin-embedded sections were deparaffinized, rehydrated, and subjected to antigen retrieval. After blocking with 3% BSA for 30 min at room temperature, sections were incubated overnight at 4 °C with primary antibody anti-F4/80 (1:1000, Cat# GB11027, Servicebio, Wuhan, China), anti-γ-H_2_AX (1:100, Cat# GB111841, Servicebio, Wuhan, China), anti-TUNEL (1:50, Cat# G1504, Servicebio, Wuhan, China) and anti-Ki-67 (1:500, Cat# GB151499, Servicebio, Wuhan, China). After washing, sections were incubated with fluorescent secondary antibodies. Nuclei were counterstained with DAPI (1:1000, Cat# G1012, Servicebio, Wuhan, China) for 10 min at room temperature. Fluorescent images were visualized by ortho-fluorescent microscopy (NIKON ECLIPSE C1, Nikon Corporation, Tokyo, Japan). Histological and immunofluorescence analyses were performed in a blinded manner whenever possible.

### 4.9. Lipids and Biochemical Measurement

To assess hepatic triglycerides (TG) content, 50 mg of fresh liver tissues were homogenized in buffer (50 mM Tris-HCl (pH 8.0), 0.25% sucrose, 1 mM EDTA). Lipids were extracted using a chloroform: methanol (2:1, *v*/*v*), followed by vortex mixing and incubation at room temperature as previously described [71,72]. Extracted lipids were dried and resuspended in isopropanol. TG content was quantified enzymatically using the Triglyceride assay kit (Cat# 290-63701, Fujifilm Wako Pure Chemical Corporation, Osaka, Japan). Serum levels of alanine aminotransferase (ALT), aspartate aminotransferase (AST), TG, total cholesterol (TC), low-density lipoprotein cholesterol (LDL-C) and high-density lipoprotein cholesterol (HDL-C) were measured by a HITACHI 7020 Automatic Analyzer (Hitachi High-Tech Corporation, Tokyo, Japan) using FUJIFILM Wako reagents (Fujifilm Wako Pure Chemical Corporation, Osaka, Japan).

### 4.10. RNA-Sequencing

Total RNA was extracted from mouse livers (n = 3 per sex/age/genotype group) using QIAzol Lysis Reagent (Qiagen, Hilden, Germany). RNA concentration and quality were assessed using an Agilent 5300 Bioanalyzer, a NanoDrop ND-2000 spectrophotometer (version 1.6; Thermo Fisher Scientific, Waltham, MA, USA), and 1% agarose gel electrophoresis. Only high-quality RNA samples (A260/A280 = 1.8–2.2, A260/A230 ≥ 2.0, RQN ≥ 6.5, 28S:18S ≥ 1.5) were used for library construction. Total RNA was hybridized with a single-stranded DNA probe designed to target rRNA. After hybridization, both the rRNA and the bound DNA probe were enzymatically removed and the remaining RNA was purified using RNA Clean Beads. The purified RNA was fragmented using divalent cations to generate suitable templates for library preparation. Reverse transcription was performed using random primers to synthesize cDNA from the fragmented RNA. During the second-strand synthesis, dUTP (2′-deoxyuridine 5′-triphosphate) was incorporated, while 5′ phosphorylation and 3′ adenylation were performed simultaneously in the same reaction. Illumina-compatible adaptors were ligated to both ends of the double-stranded cDNA to allow identification and sequencing during downstream processes. The adaptor-ligated fragments were purified and size-selected using DNA Clean Beads to enrich the desired library fragments. Next, PCR amplification was performed using primers to generate the final library. Uracil-DNA glycosylase was included in the PCR mix to degrade the dUTP-containing second strand and ensure strand specificity. Rigorous quality control checks were performed to confirm the suitability of the library for sequencing [73]. Final libraries were quantified using a Qubit 4.0 Fluorometer with Qubit Assay software (version 1.0; Thermo Fisher Scientific, Waltham, MA, USA), size-selected to 300 bp, and sequenced on Illumina NovaSeq X Plus platform (Illumina Control Software version 1.2; Illumina, Inc., San Diego, CA, USA) (2 × 150 bp, >30 million reads per sample) following the manufacturer’s instructions. Comprehensive RNA-seq quality metrics were summarized in Supplementary Table S2. Differentially expressed genes (DEGs) were identified using DESeq2 R Package (version 1.36.0) (log_2_|Fold change| ≥ 1, adjusted *p* < 0.05). Kyoto Encyclopedia of Genes and Genomes (KEGG) pathway enrichment was performed using SciPy package (version 1.14.1) runing in Python enviroment (version 3.9.12, Python Software Foundation, Wilmington, DE, USA).

### 4.11. Statistical Analysis

Data are presented as mean ± SEM from ≥3 biologically independent replicates. Statistical analyses were performed using GraphPad Prism (version 9.5.1, GraphPad Software, San Diego, CA, USA). Comparisons between two groups were performed using an unpaired two-tailed Student’s *t*-test. Multi-group comparisons were performed using one-way ANOVA with genotype as the factor, or two-way ANOVA (sex and genotype) with specified multiple comparison tests in corresponding figure legends. We reported the *p* values for main effects (sex, genotype) and interaction terms in Supplementary Table S3. *p* < 0.05 was considered significant. No animals or data points were excluded from the analyses unless technical failure occurred. The exact sample size (*n*) for each experiment is indicated in the corresponding figure legends.

## Data Availability

The data presented in this study are available on request from the corresponding authors. The data are not publicly available due to privacy.

## Supplementary Materials

The following supporting information can be downloaded at: https://www.mdpi.com/article/10.3390/ijms27157039/s1.
