# Supplementary material for: Hepatic SIRT6 Deficiency Accelerates Female-Specific Aging Through SULT1E1-Mediated Estrogen Depletion
Source: Int J Mol Sci. 2026 Aug 5;27(15):7039. doi: 10.3390/ijms27157039 (PMC13467427; doi:10.3390/ijms27157039)
Supplement: Supplementary file 1 [file ijms-27-07039-s001.zip › Supplementary Figures S1-S6.pdf]

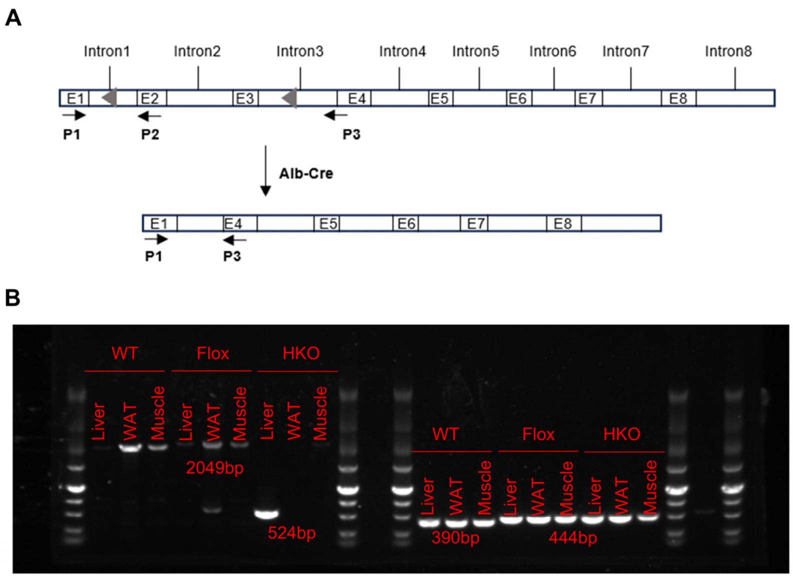

Supplementary Figure S1. Generation of hepatocyte-specific *Sirt6* knockout mice.

A. Schematic of *Albumin*-Cre recombination. The conditional *Sirt6*$^{fl/fl}$ allele contains loxP sites (triangles) flanking exons 2-3. Cre-mediated excision generates the null allele (*Sirt6*$^{\Delta hep}$, hepatocyte-specific *Sirt6* knockout, HKO-*Sirt6*). **B.** PCR validation of tissue-specific deletion. *Sirt6* is deleted in liver tissue, but not in white adipose tissue (WAT) or skeletal muscle: Primers P1 (5'-GCTAATGGGAACGAGACCAA-3') and P3 (5'-GCGTCCACTTCTTTCCTG-3') are used to generate a 524-bp recombinant band in liver, and a 2049-bp non-recombined product in WAT and skeletal muscle. Primers P1 and P2 (5'-ACCCACCTCTCTCTCCCTAAA-3') amplify both the wild type and floxed *Sirt6* alleles. The floxed allele yields a PCR product of 444 bp, while the wild-type allele produces a product of 390 bp.

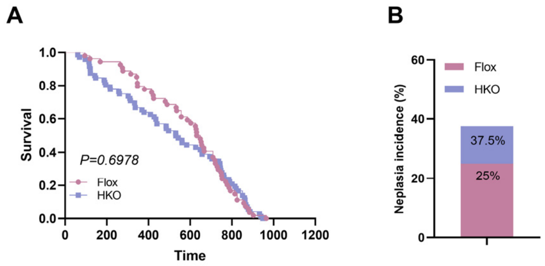

Supplementary Figure S2. Sex-pooled analysis of survival and neoplasm incidence in HKO-*Sirt6* mice.

A. Kaplan-Meier survival curves of Flox control (n = 23 females, n = 31 males) and HKO (n = 36 females, n = 36 males) mice (Log-rank test *P* = 0.698; Gehan-Breslow-Wilcoxon test *P* = 0.227). **B.** Neoplasm incidence in aged cohorts (n = 16 mice per genotype). Significance between genotypes was assessed using a two-tailed Fisher's exact test (*P* = 0.220).

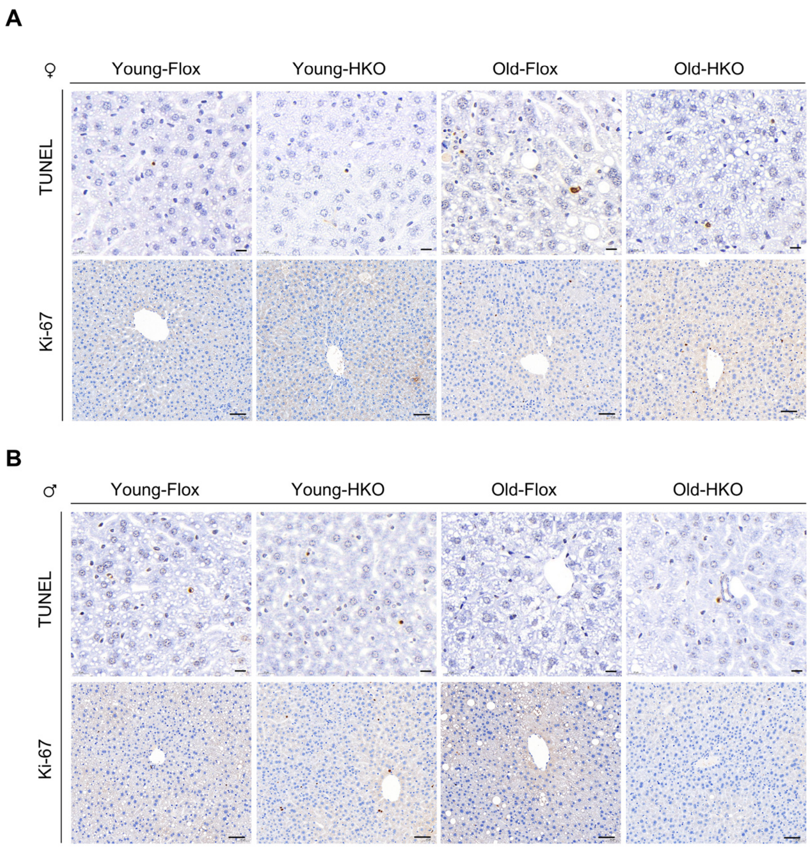

Supplementary Figure S3. Hepatic apoptosis and proliferation in aged HKO-*Sirt6* mice.

A-B. Representative immunohistochemistry images of TUNEL staining (apoptosis, top) and Ki-67 staining (proliferation, bottom) in liver sections from female **(A)** and male **(B)** mice (n ≥ 3 mice per genotype/sex/age). Scale bar: 50 μm.

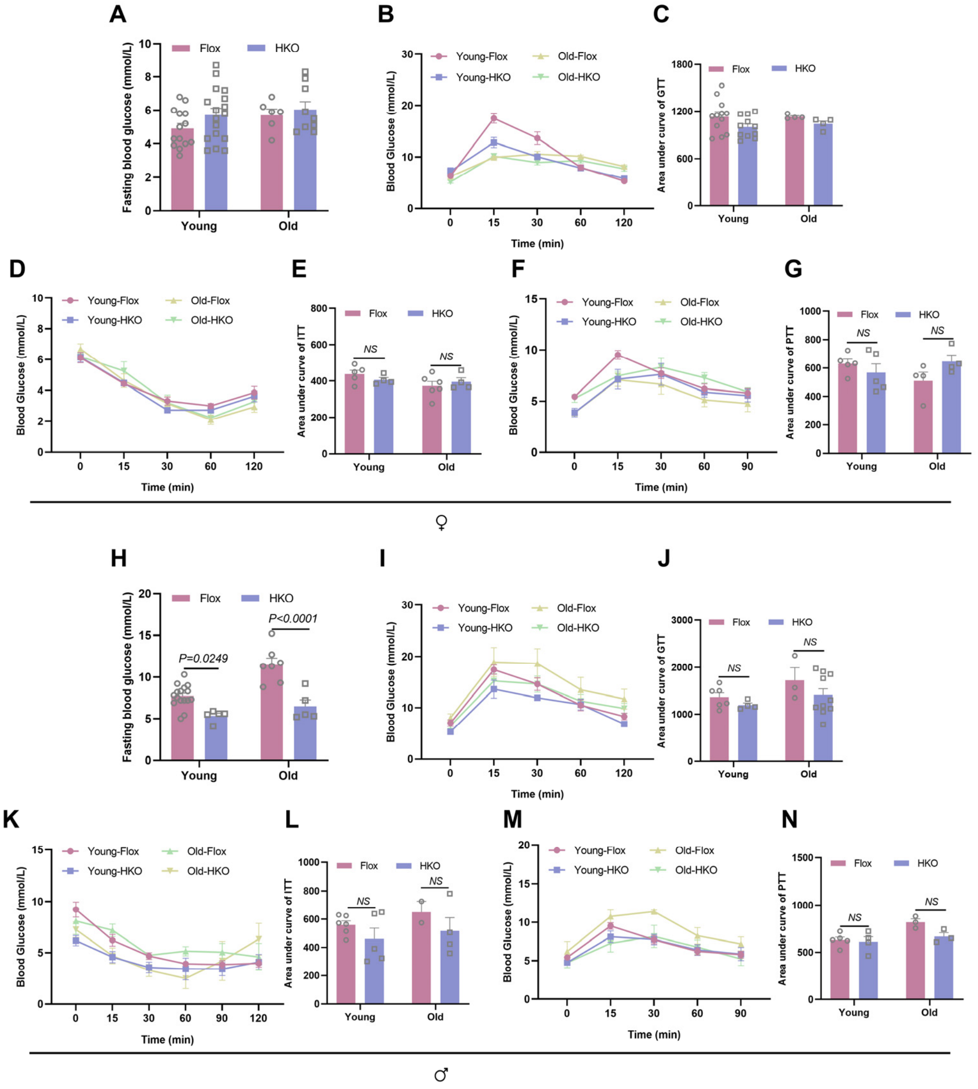

Supplementary Figure S4. Glucose homeostasis in hepatocyte-specific *Sirt6*-deficient mice of both sexes.

Glucose profiling in females (A-G, n = 4-12 per group) and males (H-N, n = 3-12 per group) across the age cohorts. Fasting blood glucose levels (**A, H**). Oral glucose tolerance test (GTT) with area under the curve (AUC) after a 16-h fast **(B-C, I-J)**. Insulin tolerance test (ITT) with AUC after a 4-h fast **(D-E, K-L)**. Pyruvate tolerance test (PTT) with AUC after a 16-h fast **(F-G, M-N)**. Data are presented as mean ± SEM. Statistical analyses were performed using two-way ANOVA with Tukey's post hoc analysis.

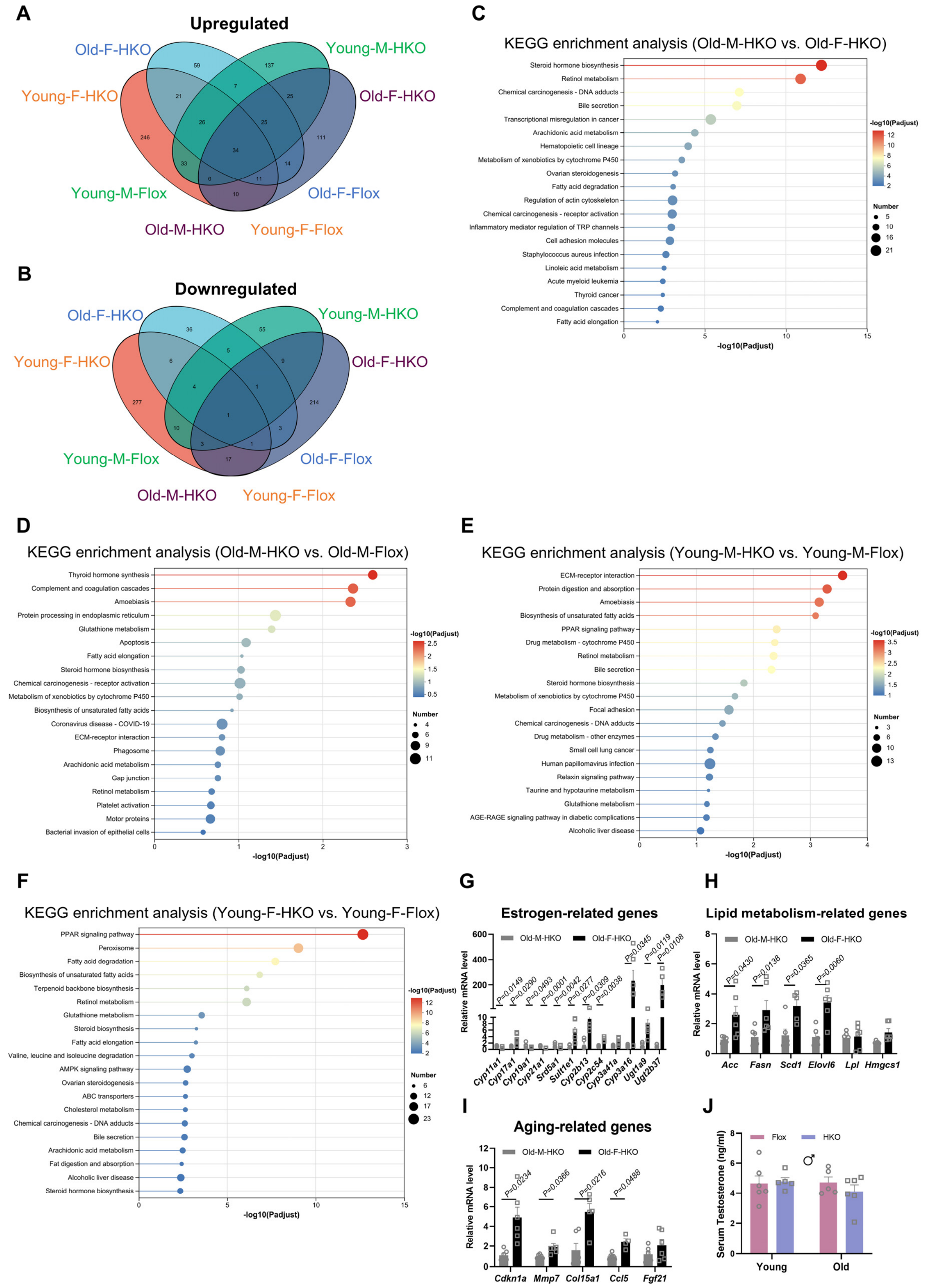

Supplementary Figure S5. Stratified hepatic transcriptomic profiling and sex-

layered validation in Flox- and HKO-*Sirt6* mice.

A-B. Venn diagrams of co-regulated differentially expressed genes (DEGs; $|log_2FC| \geq 1$, adj. $P < 0.05$) between Flox and HKO mice across age and sex. Upregulated genes **(A)**. Downregulated genes **(B)**. Group comparisons: Young-Female-Flox vs. -HKO; Old-Female-Flox vs. -HKO; Young-Male-Flox vs. -HKO; Old-Male-Flox vs. -HKO (n = 3 per group). **C-F.** Top enriched KEGG pathways ranked by $-log_{10}$(adjusted. *P*): Aged male HKO and female HKO mice **(C)**, aged male HKO and Flox mice **(D)**, young male HKO and Flox mice **(E)** and young female HKO and Flox mice **(F)** (n = 3 for each group). **G-I.** Sex-divergent qPCR validation of estrogen **(G)**, lipid metabolism **(H)**, and ageing-related signaling **(I)** in aged male HKO compared with female HKO mice (n = 6 per group). **J.** Serum testosterone levels were measured by ELISA in male mice (n = 5-6 per group). Data are presented as mean ± SEM. *P* values were determined by two-way ANOVA with Sidak's multiple comparisons test.

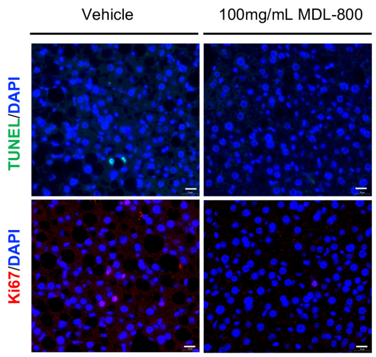

Supplementary Figure S6. Effects of MDL-800 on hepatic apoptosis and proliferation in HFD-fed aged female mice.

Representative immunofluorescence staining images of TUNEL (green, top) and Ki-67 (red, bottom) in liver sections from aged female mice treated with vehicle or MDL-800 (100 mg/kg/day for 8 weeks). Nuclei were counterstained with DAPI (blue) (n = 4-5 per group). Scale bar: 20 μm.
